# Role of platelet indices as diagnostic and predictive biomarkers for comorbidity of diabetes and metabolic syndrome in southern Ethiopia: A comparative cross-sectional study

**DOI:** 10.1371/journal.pone.0277542

**Published:** 2022-11-11

**Authors:** Kassahun Haile, Rebie Kedir, Abebe Timerga, Ayenew Mose, Mesay Arkew

**Affiliations:** 1 Department of Medical Laboratory Science, College of Medicine and Health Sciences, Wolkite University, Wolkite, Ethiopia; 2 Department of Biomedical Science, College of Medicine and Health Sciences, Wolkite University, Wolkite, Ethiopia; 3 Department of Midwifery, College of Medicine and Health Sciences, Wolkite University, Wolkite, Ethiopia; 4 School of Medical Laboratory Science, College of Health and Medical Sciences, Haramaya University, Harar, Ethiopia; Bolu Abant İzzet Baysal University: Bolu Abant Izzet Baysal Universitesi, TURKEY

## Abstract

**Background:**

Metabolic syndromes (MetS) and diabetes mellitus (DM) comorbidity is a growing major global public health problem with huge morbidity and mortality. It is a pro-inflammatory and prothrombotic disorder characterized by alteration of platelet indices and increased platelet activation, however, the tendency to use them in diagnosis is not yet fully evaluated in our context and there is limited evidence on the role of platelet indices in predicting and differentiating DM+MetS comorbidity in Ethiopia. Thus, this study aimed to evaluate platelet indices in HC, DM, and DM+MetS, and to determine their role in the prediction of DM+MetS comorbidity risk and the distinction between DM+MetS and DM or healthy persons in southwest Ethiopia.

**Method and materials:**

A comparative cross-sectional study was conducted in Wolkite University specialized hospital from March to August 2021. A total of 336 study participants (112 healthy controls (HC), 112 DM, 112 DM+MetS) was included in this study. Anthropmetric data were measured and the venous blood sample was collected to determine platelet indices, lipid profiles, and blood glucose levels. The SPSS version 21 statistical software was used to perform receiver operating curve (ROC), one-way ANOVA, and independent T-test analysis. The p-value for statistical significance was set at <0.05.

**Result:**

In the present study, we found a significant difference in the mean value of PLT, MPV, and PDW between DM+MetS, DM, and HC. A statistically significant difference in the mean value of MPV and PDW was observed between HC and DM+MetS as well as DM and DM+MetS (p-value<0.001). At the cutoff value of 9.65fl with a sensitivity of 81.3% and a specificity of 67.9%, MPV differentiates DM+MetS from HC with an AUC of 0.859. MPV can differentiate DM+MetS from DM at a cutoff value of 10.05fl with sensitivity, specificity, and an AUC of 67.9%, 65.2%, and 0.675, respectively. At the cutoff value of 9.65fl with a sensitivity of 69.6% and a specificity of 67.9%, MPV differentiates DM from HC with an AUC of 0.747. The best platelet parameter identified in this study for predicting the presence of DM+MetS comorbidity was MPV (AUC=0.859; 95%CI=0.81-0.90).

**Conclusion:**

In this study, a significant difference in the mean value of PLT, MPV, and PDW was found between DM+MetS, DM, and HC. The mean value of platelet indices showed significant increases in DM+MetS patients in comparison to HC and DM. MPV has been identified as a good potential marker to predict DM+MetS comorbidity and to differentiate DM+MetS comorbidity from the HC or DM. Our results show that MPV could be a good hematological marker to differentiate DM+MetS comorbidity from the HC or DM, and may offer supportive information for early diagnosis, prevention, and control. Thus, the findings of this study should be taken into account for the prevention and control of DM+MetS comorbidity.

## Introduction

Metabolic syndrome is an increasingly important global public health issue affecting both developed and developing nations [1–4]. The studies revealed 34.1%, 33.3%, 21.66%, 37.05%, 19.1%, 43.83%, 20.6% and 34.2% burden of MetS in Brazil [5], Finland [6], Indonesia [7], Iran [8], Uganda [9], Ghana [10], Malaysia [11], and Ethiopia [12], respectively. According to ATP III guideline [13], MetS is defined as the presence of three or more of the following conditions: waist circumference(WC) ≥102 cm in men or ≥88 cm in women; blood pressure (BP) ≥130/≥85 mm Hg; fasting blood glucose (FBS) ≥126 mg/dl; triglyceride (TG) ≥150 mg/dl and low high-density lipoprotein (HDL) <40 mg/dl in men or <50 mg/dl in women. Studies demonstrated a high burden of MetS in diabetic patients and DM+MetS comorbidity as an emerging public health problem that needs attention [10, 13–15]. Studies indicate a burden of MetS of 90.6%, 60.4%, 70.3% and 59.62% among diabetic patients in Ghana [16], Cameroon [17] and Ethiopia [18], respectively. Another study found that two out of three Type 2 diabetic (T2DM) patients in sub-Saharan African countries had MetS [19]. Additionally, MetS comorbidity with DM is considered a major risk factor for cardiovascular disease (CVD) and mortality in patients with DM [15, 16]. Early prediction of the risk of DM+MetS comorbidity is absolutely important for DM and MetS patient management as well as for CVD prevention, decelerating disease progression, and mortality [10, 17]. However, due to the lack of established standard screening methods, the effectiveness and reliability of MetS screening in a DM patient are not convincing, and standards for MetS diagnosis are likely to vary substantially [13, 20–22]. In addition, MetS diagnosis and determination need measurements of different parameters at a time that needs sophisticated materials and are easily affected by different factors [13, 20]. It is therefore essential to discover a biomarker that is accessible, reliable, and cost-effective from laboratory reports for early prevention and control of MetS+DM comorbidity.

It is underlined that hematological indices tend to be exceptionally basic, fast, and cost-effective tools, particularly in developing countries like Ethiopia with constrained assets to distinguish early vascular complications and predict comorbidity risk. Metabolic syndrome and diabetes are pro-inflammatory and prothrombotic disorders characterized by alteration of platelet indices and increased platelet reactivity [23–25]. The evidence relates platelet activation (PLT) to MetS and DM [3, 4, 21, 25], however, the tendency to use them in diagnosis is not yet fully evaluated in our context. Platelet indices such as mean platelet volume (MPV), platelet crits (PCT), and platelet distribution width (PDW) are the indicators of increased platelet activity [23, 24] and have been identified as potential new biomarkers of disease severity and comorbidity [25]. Studies have shown that changes in platelet counts (PLT) and MPV are independently associated with the presence and severity of COVID-19 [26], tuberculosis [27], malaria [28], and hypertension [29]. In addition, MPV has demonstrated good prognostic and diagnostic value in predicting the risk of TB+DM comorbidity, and significantly distinguishes TB+DM comorbidity from TB or DM [27]. However, its role in the prediction of DM+MetS comorbidity risk and the distinction between DM+MetS and DM or healthy persons is not adequately described.

Few studies have demonstrated relations between hematological parameters and MetS [30–33], with a controversial conclusion. An increase in the PLT, PCT, and PDW were observed in older adults with MetS compared to healthy control [32]. The elevated level of MPV and the PDW significantly predict the risk of vascular complications in DM patients [33, 34]. A significantly higher level of MPV, PDW, and P-LCR was seen in T2DM patients with microvascular complications [33, 35, 36]. However, the role of platelet indexes in the prediction of DM+MetS comorbidity risk and the distinction between DM+MetS and DM or HC is not well known in Ethiopia. Thus, this study aimed to evaluate platelet indices in HC, DM, and DM+MetS, and to determine their role in the prediction of DM+MetS comorbidity risk and the distinction between DM+MetS and DM or HC in southwest Ethiopia.

## Method and materials

### Study area

The study was conducted in Wolkite University specialized hospital, which is located 158km far from the capital city of Ethiopia, Addis Ababa. The hospital was found in the Gubriye sub-city in the Gurage zone, Southern Nations, Nationalities and Peoples Regional State (SNNPR), Ethiopia. The hospital provides services for more than 1.2 million people living in the Gurage zone and the neighboring area. The study was conducted on DM patients attending their follow-up at the chronic illness clinic of the Wolkite University specialized hospital.

### Study design and period

A facility-based comparative cross-sectional study was employed from March to August 2021.

### Sample size determination and sampling technique

The sample size was determined by two populations’ mean formulae using G-power version 3.1 by considering the following assumptions: 95% confidence interval (two-sided), 80% power (Z_1-β_), and 1:1 ratio of cases to control group. Sample size (n) = (SD1^2^+SD2^2^) (Z_1-α_ + Z_1-β_)^2^/(M1-M2)^2^. Where, M1and SD1are the mean and standard deviation of group 1 respectively, and M2 and SD2 are the mean and standard deviation of group 2 respectively.

Taking the mean and standard deviation (SD) of MPV for DM patients and DM+ MetS groups from the previous study [36], 8.81 and 1.6 for DM patients, whereas 9.34 and 1.57 for the DM+ MetS group. We got a total sample size of 224 (112 DM, 112 DM+MetS). A total of 112 individuals were also recruited as healthy controls (HC) who had free of DM and MetS. All consecutively identified study participants who had met inclusion criteria were included in the study. Inclusion criteria include; ages≥18 years, diagnosed with DM based on WHO criteria [FBS ≥ 7.0 mmol/L(126 mg/dl) or 2-hour post-load plasma glucose ≥ 11.1 mmol/L(200 mg/dl) or Hba1c ≥ 48 mmol/mol(6.5%)] [2], diagnosed with MetS based on ATP III guideline [presence of 3 or more of the following conditions: WC ≥102 cm in men or ≥88 cm in women; BP ≥130/≥85 mmHg; FBS ≥126mg/dl; TG≥150 mg/dl and low HDL-C <40 mg/dl in men or <50 mg/dl in women] [13], DM+MetS diagnosed DM patients along with MetS. Study participants who had bleeding manifestations, pregnancy, and previous medical history of chronic diseases that potentially affect platelet parameters, the habit of smoking cigarettes, and alcohol consumption were excluded from the study.

### Data collection and laboratory procedures

#### Socio-demographic data collection

The data on the socio-demographic characteristics (age, gender, residence) were collected through interviews by trained nurses using a structured questionnaire developed from the related literature [30, 37].

#### Anthropometric data collection

Waist circumference was measured by stretch-resistant tape at the midpoint between the lower margin of the least palpable rib and the top of the hip or minimal waist. The cut-off value of WC≥102cm for men and ≥88 cm for women were used to indicate central obesity [13]. Body weight was measured using portable weighing scales to the nearest 0.1kg and the participant was weighed without shoes and wearing very light indoor clothing. Whereas standing height without shoes was measured by an adjustable wooden measuring board and then body mass index (BMI) was calculated as weight in kilogram divided by the square of height in meter (kg/m2) [20].

#### Blood pressure measurement

Blood pressure (BP) was measured from the right upper arm using a mercury sphygmomanometer after the participants had rested for more than 5 minutes and then reported by millimeter mercury (mmHg). Interpreted as systolic blood pressure (SBP; ≥130 mmHg) or diastolic blood pressure (DBP; ≥85mmHg) to indicate hypertension [20].

#### Blood sample collection

After obtaining written informed consent from the study participants, the vein of the antecubital fossa of the forearm arm was disinfected with 70% alcohol, and a tourniquet was applied, then after the required amount of blood had been collected, the tourniquet was released.

A total of 8ml venous blood sample was collected from each study participant by a trained laboratory technologist by vacutainer tube method in two different test tubes; 4ml in a standard serum separator tube without anticoagulant for serum glucose and lipid profile analysis, and 4ml in an EDTA tube for platelet parameter determination. The blood sample collected in a serum separator tube stayed for 30 minutes at room temperature; then serum was separated by centrifugation at 4000 rpm for 5 minutes using a Rotanta-960 centrifuge. The separated serum was kept under -20°c deep freeze until biochemical analysis was performed.

#### Lipid profile and glucose analysis

Fasting blood glucose and lipid profiles (total cholesterol, high-density lipoproteins, low-density lipoprotein, and triglycerides) were determined by ABX Pentra automated analyzer (Horiba ABX SAS, Montpellier, France). Lipid profiles were measured by the enzymatic colorimetric test principle, and a series of coupled enzymatic reactions were carried out to form a colored chromogenic complex. The absorbance of the colored dye was measured at a fixed wavelength and is proportional to the concentration of the lipid profile in the sample. The enzymatic method was used to determine glucose, glucose was measured by the PAP-CP, enzymatic photometric method, and then finally absorbance was measured.

#### Hematological parameter analysis

Four milliliters of venous blood samples were collected in an EDTA tube and hematological parameters were determined by Sysmex XP-300 automated analyzer. Complete blood counts were measured by the electrical impedance principle, cell counting is based on the detection and measurement of changes in electrical resistance (pulses) produced by cells as they traverse a small aperture. The number of pulses produced by the cell is proportional to the number of cells counted.

### Data analysis and interpretation

After checking for completeness and clarity, data were entered, processed, and analyzed by SPSS version 21 statistical software (SPSS Inc., Chicago, IL). Data were presented by using descriptive statistics, tables, and figures. The Kolmogorov-Smirnov test was used to check the normal distribution of the data and all data were normally distributed. The differences between data across the groups (HC, DM, DM+MetS) were analyzed using the chi-squared test, one-way ANOVA, and independent T-test. The independent T-test was used to analyze continuous data and the chi-square test was used to analyze categorical variables. A receiver operating curve (ROC) was performed to determine the sensitivity, specificity, area under the curve (AUC), and a cutoff value for a given PLT parameter (PLT, MPV, and PDW) in discriminating the presence or absence of DM and DM+ MetS comorbidity. The p-value for statistical significance was set at <0.05.

### Ethical considerations

Ethical clearance was obtained from Wolkite University Ethical Review Committee. After explaining the purpose and procedures of the study, written informed consent was obtained from each study participant, the confidentiality of their data was kept and they were assured that only aggregate data will be reported. All necessary findings of the participant were also communicated to their respective physician for proper management.

## Results

### Basic characteristics of study participants

A total of 336 study participants (112 HC, 112 DM, 112 DM+MetS) were enrolled in this study. The total number of males and females among the three groups was 167 (49.7%) and 169 (50.3%) respectively. The mean age of the study participants was 49.6±16.0, 50.5±15.2, and 51.1±13.2 years in HC, DM, and DM+MetS groups, respectively. In the present study, there were no statistically significant differences were observed across the three groups in age, gender, and residency (**Table 1**).

**Table 1:** Basic characteristics of study participants in the three groups at Wolkite University specialized hospital from March to August 2021 (n=316).

Variables	HC	DM	DM+MetS	P-value*
Age in years	49.6±16.0	50.5±15.2	51.1±13.2	0.72
Gender (Male/Female)	51/61	59/53	57/55	0.53
Residency (Urban/Rural)	64/48	66/46	54/58	0.22
FBS (mg/dl)	79.1±8.0	192.3±51.4	164.2±64.3	<0.001
BMI (kg/m^2^)	22.8±1.66	26.9±4.31	26.7±3.77	<0.001
SBP (mmHg)	102.3±12.1	124.9±10.4	144.2±19.8	<0.001
DBP (mmHg)	80.8±2.6	86.6±10.1	90.6±9.2	<0.001
TC (mg/dl)	122.9±27.8	140.8±43.2	155.4±44.9	<0.001
TG (mg/dl)	112.8±15.5	140.1±41.0	156.2±35.9	<0.001
LDL (mg/dl)	80.9±14.3	83.2±20.8	91.1±20.9	<0.001
HDL (mg/dl)	55.8±5.6	50.1±10.4	47.9±11.7	<0.001

Abbreviations

*; P-value for categorical variables (age, residence) was derived from the chi-square test and for remaining variables, it was derived from the independent t-test, BMI; body mass index, DBP; diastolic blood pressure, FBS; fasting blood glucose, HDL; high-density lipoprotein, LDL; low-density lipoproteins, SBP; systolic blood pressure, TC; total cholesterol, TG; triglyceride, kg/m^2^; kilogram per meter square, mg/dl; milligram per deciliters, mmHg; millimeter mercury.

### Comparisons of platelet indices across the groups

The mean (SD) of platelet indices was compared among the three groups (HC, DM, DM+MetS). Accordingly, statistically significant differences were observed among the three groups in the mean value of PLT, MPV, and PDW, P-value< 0.05 (**Table 2**).

**Table 2:** Comparison of platelet indices across the groups.

Parameter	HC	DM	DM+MetS	P-value*
PLT×10^9^/L	249.8±62.9	266.7±75.7	280.7±81.0	0.008
MPV(FL)	9.44±0.5	10.03±0.7	10.6±1.05	<0.001
PDW (FL)	10.4±1.3	10.7±0.8	11.8±1.7	<0.001

Abbreviations

*: P-value was derived from one-way ANOVA, DM; diabetes mellitus, HC; health control, MetS; metabolic syndrome, MPV; mean cell platelet volume in femtoliter, PDW; platelet distribution width, PLT; platelet per litter.

### Comparisons of platelet indices between the groups

The difference in the mean value of the platelet parameter was compared between the groups. Study participants who had DM were significantly higher MPV and PDW values compared to HC. Compared with the HC, changes in the PLT, MPV, and PDW were significantly increased in DM+MetS patients (280.7±81.0*×*10^9^/L, 10.6±1.05FL, 11.8±1.7FL, p-value <0.05). Compared with DM patients (10.03±0.7FL, 10.7±0.8FL), the changes in the MPV and PDW were significantly increased in DM+MetS patients (10.6±1.05FL, 11.8±1.7FL) (**Table 3**).

**Table 3:** Comparisons of platelet indices among the three groups.

Parameter	HC	DM	DM+MetS	HC VS DM P-value*	HC VS DM+MetS P-value*	DM VS DM+MetS P-value*
PLT×10^9^/L	249.8±62.9	266.7±75.7	280.7±81.0	0.07	0.002	0.18
MPV(FL)	9.44±0.5	10.03±0.7	10.6±1.05	<0.001	<0.001	<0.001
PDW (FL)	10.4±1.3	10.7±0.8	11.8±1.7	0.034	<0.001	<0.001

Abbreviations

*; P-value was derived from the independent t-test, DM; diabetes mellitus, HC; health control, MetS; metabolic syndrome, MPV; mean cell platelet volume in femtoliter, PDW; platelet distribution width, PLT; platelet per litter.

### Diagnostic value of platelet parameters for DM+MetS comorbidity

ROC curve analysis was used to determine the optimal cutoff values of PLT parameters for the prediction of DM and DM+MetS comorbidity. MPV can differentiate DM patients from healthy controls at a cutoff value ≥9.65 FL with a sensitivity of 69.6%, specificity of 67.9%, and with an AUC of 0.747 (p-value <0.001).

**Fig 1:**
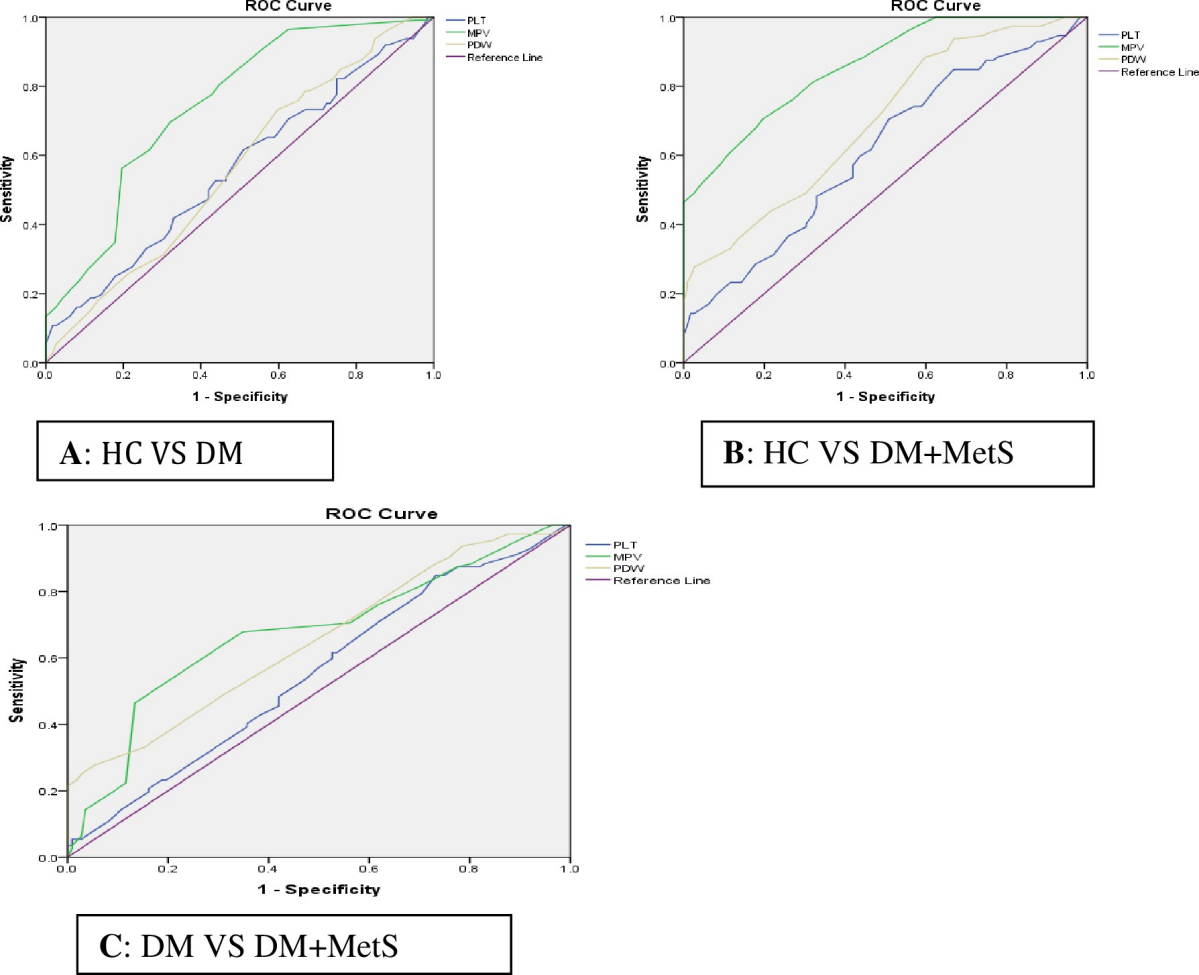
ROC curve analysis of the PLT parameter of study participants at Wolkite University specialized hospital from March to August 2021 (n=316). **A**. ROC curve for differential diagnosis of HC VS DM; **B**. ROC curve for differential diagnosis of HC VS DM+MetS; **C**. ROC curve for differential diagnosis of DM VS DM+MetS.

MPV has the largest area under the curve (AUC = 0.747; 95%CI (0.68-0.81) in a differential diagnosis of HC VS DM as well as in a differential diagnosis of HC VS DM+MetS (AUC=0.859; 95%CI (0.81-0.90) and DM VS DM+MetS (AUC=0.675; 95%CI (0.603-0.74) respectively, which showed that it is the best platelet parameter for predicting and discriminating DM+MetS comorbidity (**Table 4 and Fig 1A–1C**).

**Table 4:** Diagnostic value of platelet indices for DM and DM+MetS comorbidity, 2021.

Parameter	Sensitivity (%)	Specificity (%)	Cutoff value	AUC	95%CI	P-value
**HC VS DM**						
PLT×10^9^/L	53.6%	53.6%	≥235	0.559	0.48-0.63	0.12
MPV(FL)	69.6%	67.9%	≥9.65	0.747	0.68-0.81	<0.001
PDW (FL)	57.1%	50.9%	≥10.95	0.561	0.48-0.63	0.11
**HC VS DM+MetS**						
PLT×10^9^/L	61.6%	53.6%	≥239.5	0.613	0.54-0.68	0.003
MPV(FL)	81.3%	67.9%	≥9.65	0.859	0.81-0.90	<0.001
PDW (FL)	72.3%	50.9%	≥10.95	0.698	0.63-0.76	<0.001
**DM VS DM+MetS**						
PLT×10^9^/L	53.6%	52.7%	≥257	0.552	0.47-0.62	0.17
MPV (FL)	67.9%	65.2%	≥10.05	0.675	0.603-0.74	<0.001
PDW (FL)	49.1%	68.7%	≥11.1	0.651	0.58-0.72	<0.001

Abbreviations: AUC; area under the curve, DM; diabetes mellitus, HC; health control, MetS; metabolic syndrome, MPV; mean cell platelet volume in femtoliter, PDW; platelet distribution width, PLT; platelet per litter.

The sensitivity and specificity of PLT in the differentiation of DM+MetS patients from HC were 61.6% and 53.6% at a cutoff value ≥239.5×10^9^/L with an AUC of 0.613. The sensitivity and specificity of MPV in a differential diagnosis of HC VS DM+MetS were 81.3% and 67.9% (P-value <0.001), while, 67.9% and 65.2% in the differential diagnosis of DM VS DM+MetS (P-value <0.001). Whereas, the sensitivity and specificity of PDW in a differential diagnosis of HC VS DM+MetS patients were 72.3% and 50.9% (p-value <0.001), while 49.1% and 68.7% in the differential diagnosis of DM VS DM+MetS (p-value <0.001).

## Discussion

Different hematological biomarkers have been used in routine clinical practice as changes in these markers have served as prognostic markers of disease severity and comorbidity [23, 27, 28, 38], however, there is limited evidence on the role of platelet indices as a diagnostic and predictive biomarker of DM+MetS comorbidity in Ethiopia. Thus, this study aimed to evaluate platelet indices in HC, DM, and DM+MetS, and to determine their role in the prediction of DM+MetS comorbidity and the distinction between DM+MetS and DM or HC in southwest Ethiopia. We observed a significant difference in the average value of PLT, MPV and PDW between DM+MetS, DM and, HC. A statistically significant difference in the average value of MPV and PDW was found between HC VS DM and HC VS DM+MetS as well as DM and DM+MetS. At the cutoff value of 9.65fl with a sensitivity of 81.3% and a specificity of 67.9%, the MPV differentiates DM+MetS from HC with an AUC of 0.859. MPV can differentiate DM+MetS from DM at a cutoff value of 10.05fl with sensitivity, specificity, and an AUC of 67.9%, 65.2%, and 0.675, respectively. At the threshold value of 9.65fl with a sensitivity of 69.6% and a specificity of 67.9%, MPV distinguishes DM from HC with an AUC of 0.747. The best platelet parameter identified in this study to predict the presence of DM+MetS comorbidity was MPV (AUC=0.859; 95%CI=0.81-0.90).

In this study, the mean value for PLT, MPV and PDW demonstrated a statistically significant difference between the HC, DM and DM+MetS groups. Our conclusion was consistent with studies across the globe [30]. The possible reason that justifies the observed difference might be the effect of the disorder on the PLT parameters, as MetS and DM are considered as one of the proinflammatory and prothrombotic disorders [36], which may result in alteration of the platelet indices and contribute to the observed difference.

In our study, we found that mean levels of PLT, MPV and PDW were significantly higher in patients with DM+MetS than in healthy controls. Our finding was in agreement with the studies reported from different parts of the world [30, 36]. Ding et al indicated that a higher level of MPV was significantly associated with MetS complications [30]. Jesri et al revealed that platelet counts were significantly increased with the severity of MetS after adjusting for other covariates [39] as well as Fang et al showed that higher platelet counts were significantly associated with MetS complications [40]. Increased platelet activation and vascular reactivity secondary to insulin resistance, oxidative stress, inflammation, hyperglycemia and non-enzymatic glycosylation in patients with DM+MetS [41, 42] may contribute to an increase in platelet parameters. Contrary to our findings, a study conducted in Taiwan showed that there is no significant difference in the mean PLT between MetS and the healthy control group [41]. This could be due to the difference in sample size and haematological analyzer.

According to our findings, MPV was significantly distinguished DM+MetS comorbidity from HC at a cutoff value of 9.65fl with a sensitivity of 81.3%, specificity of 67.9%, and with an AUC of 0.859. Consequently, MPV was implicated as a good biomarker for predicting the presence of DM+MetS comorbidity in this study. This finding was consistent with the study reported in China [30], and another similar study showed that MPV and PCT significantly distinguish TB+DM comorbidity from TB or DM individuals [27]. In addition, a study conducted in Turkey suggested that elevated MPV could significantly predict advanced fibrosis in chronic hepatitis C patients at a cutoff value greater than 7.3 FL with 76% sensitivity and 59% specificity [43]. The probable reason that justifies this relationship might be due to increased platelet activation in DM+MetS patients in response to inflammatory stimuli which may lead to enlargements of platelet size and alteration of its indices. Several studies suggested that MetS is a proinflammatory and prothrombotic state and characterized by alteration of platelet indices [36]. MPV is a surrogate marker of platelet activation which refers to the mean size of platelets and is associated with a wide variety of diseases stage [24, 27, 44]. A study conducted in India revealed that MPV was significantly increased with the severity of the preeclampsia in pregnant women, and had good discriminatory value with the severity of the disease; it discriminated healthy controls and severe preeclampsia with 74.6% diagnostic accuracy at a cutoff value greater than 9.05 FL, as well as it discriminated healthy controls and non-severe preeclampsia with 69.4%, 9.05 FL, 50.0%, and 82.4% diagnostic accuracy, cutoff value, sensitivity, and specificity, respectively [45]. Another study suggested that elevated MPV should alert physicians for hypothyroidism and MPV significantly predict hypothyroidism at a cutoff value greater than 9.47 FL with 80% sensitivity and 72% specificity [46].

The mean level of PDW showed a statistically significant difference between DM+MetS patients and HC in this study. The PDW cutoff value was 10.95fl to distinguish DM+MetS patients from HC with sensitivity, specificity, and an AUC of 72.3%, 50.9%, and 0.698, respectively. Different previous studies suggested that MPV and PDW were elevated in some clinical conditions [34, 36, 41, 44]. Buch et al revealed that MPV and PDW were significantly increased in diabetic patients with complications as compared to diabetics without complications and healthy groups; also they suggested MPV and PDW as good predictive biomarkers for diabetic vascular complications [34]. Patients with DM+MetS comorbidity have a high risk of microcirculation complications, which may probably lead to platelet hyperactivity and increased platelet volume that may result in an elevated PDW. Aktas et al suggested that elevated PDW could serve as a diagnostic tool for irritable bowel syndrome and a significantly higher mean value of PDW was found in the irritable bowel syndrome groups as compared to the control group [47]. Another study revealed that the mean level of PDW showed a statistically significant difference between liver steatosis patients and control groups, and PDW values higher than 16.25% have 83% sensitivity and 57% specificity in predicting liver steatosis [48].

The mean level of PLT showed a statistically significant difference between DM+MetS patients and HC. Our finding was in agreement with a study reported from South Carolina [39] and China [49]. The pathophysiology of MetS favored by inflammation, insulin resistance, oxidative stress, and endothelial dysfunction [4], which may trigger platelet hyperactivity and may result in observed differences in the PLT count.

The mean level of MPV and PDW were significantly increased in the DM+MetS patients compared with DM patients in the present study. This finding was in line with the studies conducted in China [30, 49], and India [34, 36, 50]. MPV differentiated DM+MetS patients from DM patients at a cut-off value of 10.05fl with sensitivity, specificity, and an AUC of 67.9%, 65.2%, and 0.675, respectively. These findings have been supported by the findings of previous literature. For instance, Rajagopal et al concluded that MPV levels were increased with increasing severity of hypertension and suggested it as a cost-effective diagnostic and predictive marker for hypertensive vascular complications [29]. In addition, another study revealed that MPV clearly distinguishes TB+DM comorbidity from DM or TB patients [27].

MPV is a widely used hematological marker of platelet size and activity and is associated with accelerated thrombopoiesis and an increased risk of cardiovascular diseases [24, 51]. In the present study, the mean value of MPV and PDW were significantly increased in the DM patients as compared to HC. MPV can differentiate DM patients from HC at a cutoff value of 9.65fl with a sensitivity of 69.6%, specificity of 67.9%, and with an AUC of 0.747, which was significant. Our finding was in line with studies reported from Turkey [35, 52, 53], India [34, 35, 54], and China [55]. Several studies suggest that platelet sensitivity in DM patients increases due to the action of the various platelet aggregation inductors including ADP, thrombin, and collagen as well as metabolic abnormalities associated with insulin resistance that affect the functional activity of platelet [30, 42, 56], which could likely result in an alteration of platelet indices in patients with DM.

## Conclusion

In this study, a significant difference in the mean value of PLT, MPV, and PDW was found between DM+MetS, DM, and HC. The mean value of platelet indices showed significant increases in DM+MetS patients in comparison to HC and DM individuals. MPV has been identified as a good potential marker to predict DM+MetS comorbidity and to differentiate DM+MetS comorbidity from the HC or DM. MPV can significantly differentiate patients with DM from HC. Our results show that MPV could be a good hematological marker to differentiate DM+MetS comorbidity from the HC or DM, and may offer support for early diagnosis, prevention, and control. Thus, the findings of this study should be taken into account for the prevention and control of DM+MetS comorbidity.

## Supporting information

S1 File English version questioner. (DOCX)

S2 File DM+MetS SPSS data set. (SAV)
