# Supplementary material for: Role of platelet indices as diagnostic and predictive biomarkers for comorbidity of diabetes and metabolic syndrome in southern Ethiopia: A comparative cross-sectional study
Source: PLoS One. 2022 Nov 11;17(11):e0277542. doi: 10.1371/journal.pone.0277542 (PMC9651558; doi:10.1371/journal.pone.0277542)
Supplement: S1 File — (DOCX) [file pone.0277542.s001.docx]

**English version questioner**

Code __________

| 1 | Age in years | ___________ |
| --- | --- | --- |
| 2 | Gender | 1. Female |
|  |  | 1. Male |
| 3 | Residence | 1. Urban |
|  |  | 1. Rural |
